# Diversity of myxozoans parasitizing the catfish *Rhamdia quelen* (Siluriformes: Heptapteridae), in southeastern Brazil, based on morphological and molecular evidence

**DOI:** 10.1038/s41598-022-22418-3

**Published:** 2022-10-20

**Authors:** Diego Henrique Mirandola Dias Vieira, Rodrigo Bravin Narciso, Reinaldo José da Silva

**Affiliations:** Institute of Biosciences, Division of Parasitology, São Paulo State University (Unesp), Botucatu, SP 18618-689 Brazil

**Keywords:** Microbiology, Parasitology, Parasite genetics, Zoology, Ichthyology

## Abstract

*Rhamdia quelen* is a commercially important fish in South America. During the survey of myxozoan infections in fishes from Pardo River, Paranapanema River basin, São Paulo State, Brazil, we describe three new species of *Henneguya* found parasitizing gills of *R*. *quelen*: *Henneguya bagre* n. sp., *Henneguya breviscauda* n. sp and *Henneguya novaerae* n. sp. The descriptions were based on myxospores morphology and small subunit ribosomal DNA partial sequences. Phylogenetic analysis showed a clade formed by species that parasitize Siluriformes, with *Henneguya jundiai* as a sister species of *Henneguya bagre* n. sp., *Henneguya breviscauda* n. sp and *Henneguya novaerae* n. sp. Our study indicates that the parasites infecting *R*. *quelen* belong to a lineage of myxozoans infecting Heptapteridae fishes. Using molecular and morphological characterization, the species were identified as new species for the genus *Henneguya*. Based on our analysis we recommend monitoring the presence of these parasites in farmed fishes, to analyze possible pathologies caused by them.

## Introduction

The Paranapanema basin is one of the most important basins in southeast Brazil as evidenced by several fish inventories and ecological studies performed^1,2^. Past works suggest that species compilations from the Paranapanema River basin can reach over 160 fish species^2–4^. The Pardo River is one of the main tributaries of the Paranapanema River, composing its basin, and contributing to the region's fish biodiversity. These fishes are potentially exposed to parasitic organisms in their life cycle^5^. Among the parasites are the myxozoans, which are cnidarians that can infect freshwater and marine fishes all over the world with over 2600 species identified^6,7^. In the family Myxobolidae Thélohan, 1892, *Henneguya* Thélohan, 1892 is one of the most specious genera with more than 200 described species^8^. Morphological variation of myxospores within a species or morphological similarities between species create challenges for myxosporean species identification^9–11^. Some species may have very similar myxospores but are genetically distant from each other, while in other cases, genetic data may show that what appears to be morphologically distinct species are conspecific^12–15^. In this context, the use of molecular techniques is essential for genetic differentiation between species.

The catfish *Rhamdia quelen* (Quoy and Gaimard, 1824) is a species widely distributed in South and Central America, east of the Andes, and between Venezuela and northern Argentina^16^. It is popularly known as “bagre”, “jundiá” or “mandi-guaru”. This fish species is widely accepted by the consumer market due to its excellent flavored meat, high resistance to cold periods, rapid growth, rusticity regarding handling, easy adaptation to artificial feeding, and lack of intramuscular spines^17,18^, representing about 2% of the total fish produced by the Brazilian aquaculture sector^19^, being very important for fish farming in southern Brazil^20^. Recently Grigio and Meurer^18^ proposed *R*. *quelen* as an alternative in recirculation systems to the species *Oreochromis niloticus* (Linnaeus, 1758), which is currently the most consumed species in Brazil. This catfish has nocturnal habits with a preference for quiet and deep river sites^21^ with large pebbles or with submerged trunks^22^. Females reach greater lengths and weights than males, with 66.5 cm total length (TL) being the asymptotic length of females and 52 cm TL of males, reaching more than 3 kg^21^.

Three *Henneguya* spp. have already been described in *R*. *quelen* in Brazil: *Henneguya jundiai* Negrelli, Vieira, Tagliavini, Abdallah and Azevedo, 2019 parasitizing the gills arches of *R*. *quelen* from the Jacaré-Pepira river, São Paulo state; *Henneguya rhamdia* Matos, Tajdari & Azevedo, 2005 parasitizing the gill filaments of *R*. *quelen* from Peixe Boi River, Pará state, Brazil; and *Henneguya quelen* Abrunhosa, Sindeaux-Neto, Hamoy & Matos, 2018 infecting the kidneys of *R*. *quelen* from Paracauari River, Marajó Island, Para state^23–25^.

The present study describes the morphology and phylogenetic relationships of three new species of myxozoans parasites found in the gills of the siluriform fish, *R*. *quelen*, in the Pardo River, Paranapanema River basin, São Paulo state, Brazil.

## Results

In total, 20 specimens of *R*. *quelen* were collected (7 females, 13 males, 15.4–29.5 cm TL, 60.1–480.0 g). Although equal efforts were made in all localities, the number of captured hosts was different between the locations. Eleven specimens were collected in locality 1, two specimens in locality 2, and seven specimens in locality 3. Plasmodia of *Henneguya* spp. were observed in gill filaments of 11 specimens (55% prevalence). Specific prevalence can be seen in Table 1. No other internal or external organs were parasitized by myxozoans. The description of the new species of *Henneguya* based on morphological and ssrDNA sequencing data is presented.

**Table 1:** Prevalence of each new *Henneguya* species found parasitizing the gills of *Rhamdia quelen* at different collection localities.

Species	Locality 1	Locality 2	Locality 3
*Henneguya novaerae* n. sp.	54.5% (6 of 11)	–	42.8% (3 of 7)
*Henneguya bagre* n. sp.	–	–	28.6% (2 of 7)
*Henneguya breviscauda* n. sp.	–	50.0% (1 of 2)	–

**Figure 1:**
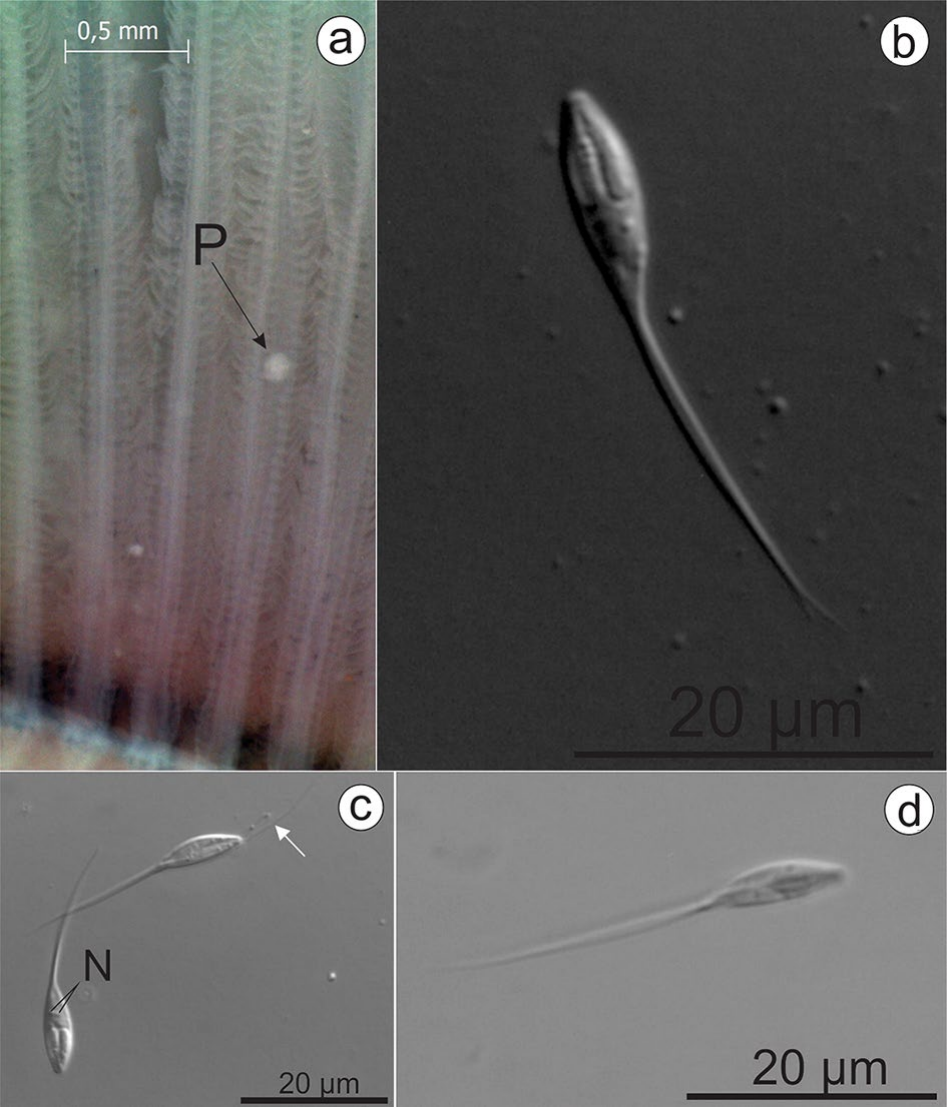
(**a**) Gills of *Rhamdia quelen*, collected from the Pardo River containing plasmodium (P) between the gill filaments. (**b**) Mature myxospore of *Henneguya novaerae* n. sp. found parasitizing the gills in front view, showing two equally-sized elongated polar capsules located side by side at the previous pole. (**c**) Mature myxospores of *Henneguya novaerae* n. sp. Note the nucleus (N) and the extruded tubule (white arrow). (**d**) *Henneguya novaerae* n. sp. myxospore in side view.

**Figure 2:**
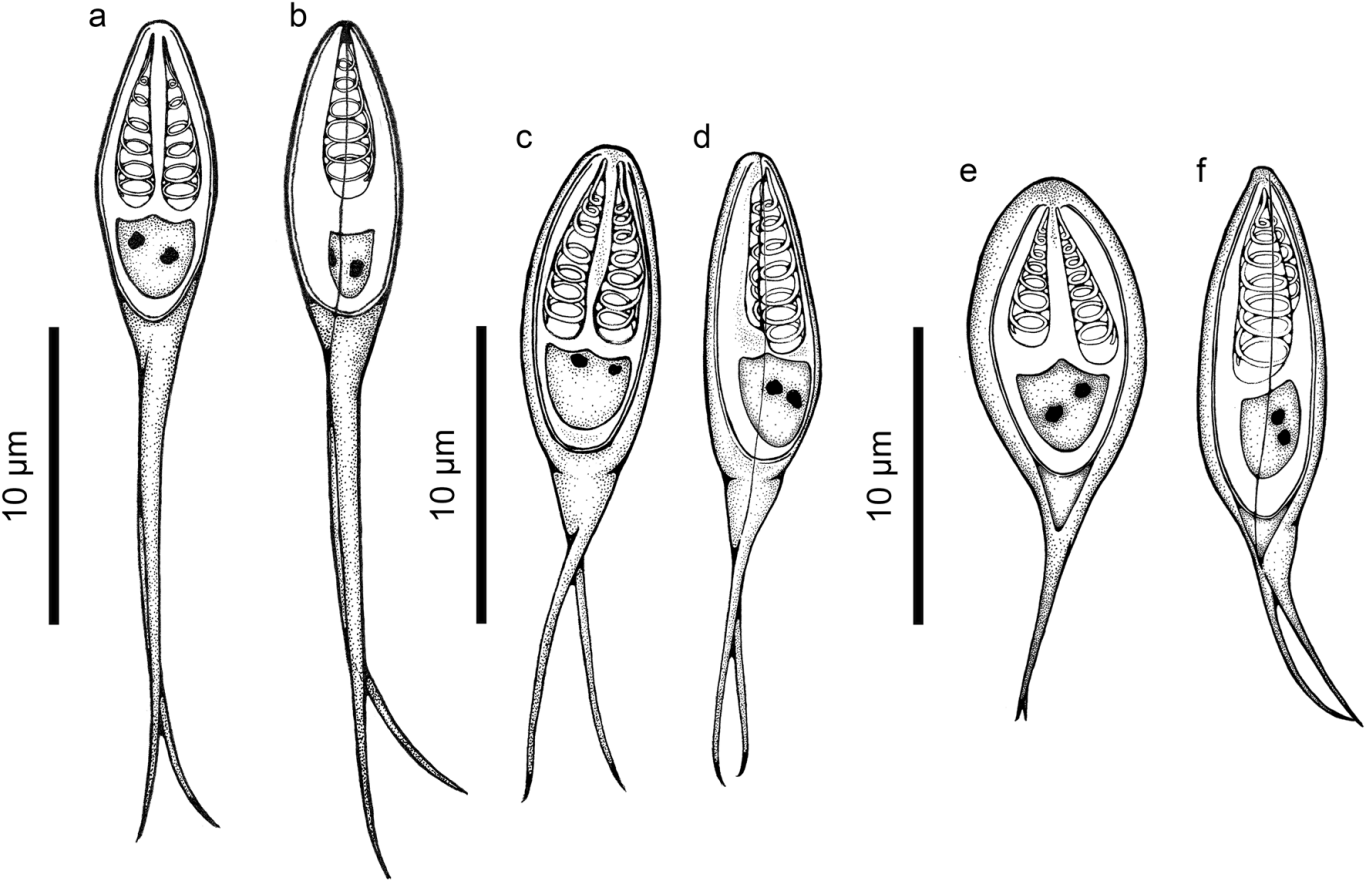
(**a**)**–(f**) Line drawings of new *Henneguya* spp. (**a**) Front view drawing of *Henneguya novaerae* n. sp. (**b**) Side view drawing of *Henneguya novaerae* n. sp. (**c**) Front view drawing of *Henneguya bagre* n. sp. (**d**) Side view drawing of *Henneguya bagre* n. sp. (**e**) Front view drawing of *Henneguya breviscauda* n. sp. (**f**) Side view drawing of *Henneguya breviscauda* n. sp.

***Henneguya novaerae* n. sp.** (Figs. 1 and 2a,b)

Type-host: *Rhamdia quelen* (Quoy & Gaimard, 1824) (Siluriformes, Heptapteridae).

Type-locality: Pardo River, Nova Era Resort, municipality of Botucatu, São Paulo State, Brazil (22° 58′ 40.03″ S; 48° 30′ 51.53″ W).

Other locality: Pardo River, municipality of Salto Grande, São Paulo State, Brazil (22° 54′ 15.9″ S; 49° 57′ 06.5″ W).

Site of infection: histozoic, gill filaments (interfilamental type).

Type-material: A glass slide with myxospores (hapantotype) was deposited in the collection of the Instituto Nacional de Pesquisa da Amazônia (INPA), Brazil (Num. INPA91). The ssrDNA partial sequences were deposited in GenBank with accession numbers OP070158-OP070160.

Etymology: The specific name is derived from the name of the locality (Nova Era Resort) where the host was collected.

Zoobank registration code: urn:lsid:zoobank.org:pub:D6824583-AAD4-4FC8-B67F-40233059FDF2.

Plasmodia: whitish, round and small, measuring about 0.1 mm. Observed in the primary filaments of the gills, occupying an interlamellar position. Average one plasmodia per parasitized fish.

Myxospores: The morphological data are based on the observation of myxospores isolated from a single plasmodium. The myxospores found were elongated and ellipsoidal in frontal view. Two elongate, elliptical shell valves with two long tapering caudal appendages. Average measurements: total length 24.9 ± 0.9 (23.3–26.8) µm, body length 10.7 ± 0.5 (10.1–11.6) µm, body width 3.8 ± 0.3 (3.2–4.2) µm, caudal appendages length 14.7 ± 1.4 (11.5–16.1) µm. Myxospores were biconvex in the lateral view with a thickness of 3.5 ± 0.3 (3.1–4.0) µm. The polar capsules were equal in size and measured 4.8 ± 0.4 (3.8–5.5) µm in length and 1.3 ± 0.2 (1.1–1.7) µm in width, occupying about half of the sporoplasm. Six coils in the polar tubules present inside the polar capsule perpendicular to the longitudinal axis of the polar capsule were observed. Binucleated sporoplasm and symmetrical valves.

**Figure 3:**
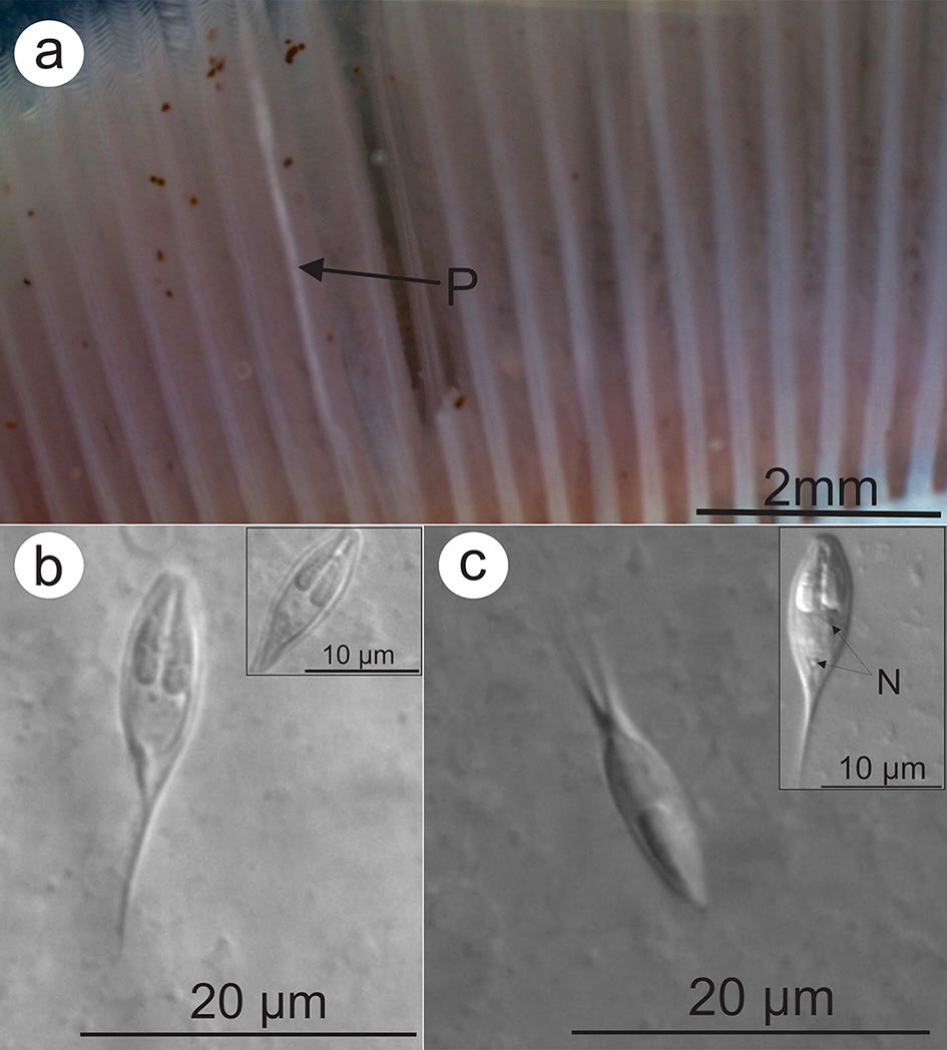
(**a**) Gills of *Rhamdia quelen*, collected from the Pardo River containing plasmodium (P) inside the gill filaments. (**b**) Mature myxospore of *Henneguya bagre* n. sp. found parasitizing the gills in front view. In highlight, the two polar capsules of equal size, elongated, showing 7 turns of the polar tubules inside it. (**c**) *Henneguya bagre* n. sp. myxospore in side view. In highlight note the binucleated sporoplasm (N).

***Henneguya bagre* n. sp.** (Figs. 2c,d and 3)

Type-host: *Rhamdia quelen* (Quoy and Gaimard, 1824) (Siluriformes, Heptapteridae).

Type-locality: Pardo River, municipality of Salto Grande, São Paulo State, Brazil (22° 54′ 15.9″ S; 49° 57′ 06.5″ W).

Site of infection: histozoic, gill filaments (intrafilamental type).

Type-material: A glass slide with myxospores (hapantotype) was deposited in the collection of the Instituto Nacional de Pesquisa da Amazônia (INPA), Brazil (Num. INPA92). The ssrDNA partial sequence was deposited in GenBank with accession number OP070161.

Etymology: The specific name is derived from the Brazilian popular name of *R*. *quelen* (bagre).

Zoobank registration code: urn:lsid:zoobank.org:pub:D6824583-AAD4-4FC8-B67F-40233059FDF2.

Plasmodia: whitish, elongated, occupying the inner region of the primary gill filament (intralamellar), measuring about 3 mm. Average one plasmodia per parasitized fish.

Myxospores: The morphological data are based on the observation of myxospores isolated from a single plasmodium. The myxospores found were elongated and ellipsoidal in frontal view. Two elongate, elliptical shell valves with two medium tapering caudal appendages. Average measurements: total length 20.9 ± 0.9 (19.2–21.8) µm, body length 11.2 ± 0.5 (10.5–12.4) µm, body width 5.1 ± 0.3 (4.6–6.1) µm, caudal appendages length 9.8 ± 0.9 (8.2–11.1) µm. Myxospores were biconvex in the lateral view with a thickness of 3.8 ± 0.2 (3.6–4.1) µm. The polar capsules were equal in size and measured 5.9 ± 0.6 (4.9–6.7) µm in length and 1.6 ± 0.1 (1.4–1.8) µm in width, occupying about half of the sporoplasm. Seven coils in the polar tubules present inside the polar capsule perpendicular to the longitudinal axis of the polar capsule were observed. Binucleated sporoplasm and symmetrical valves.

**Figure 4:**
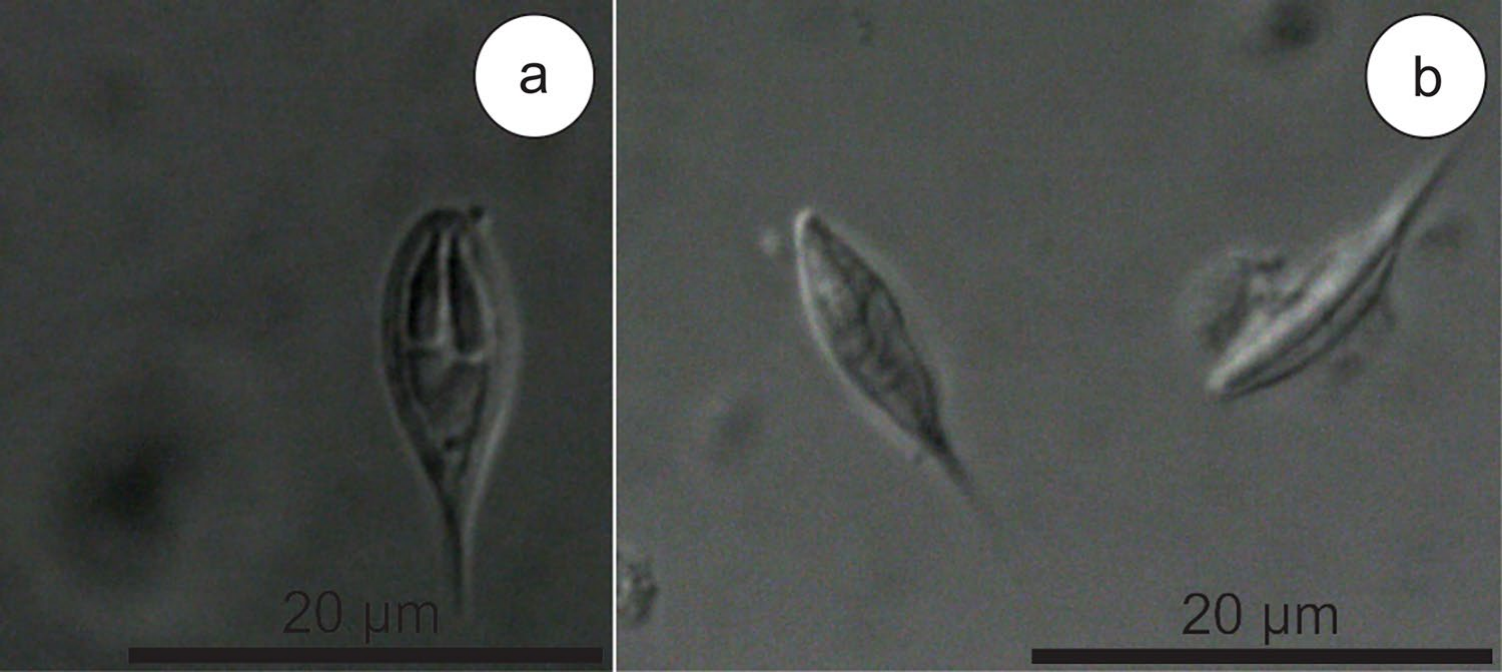
(**a**) Mature myxospore of *Henneguya breviscauda* n. sp. found parasitizing the gills in front view. Note the polar capsules in unequal size. (**b**) *Henneguya breviscauda* n. sp. myxospores in side view.

***Henneguya breviscauda* n. sp.** (Figs. 2e–f and 4)

Type-host: *Rhamdia quelen* (Quoy and Gaimard, 1824) (Siluriformes, Heptapteridae).

Type-locality: Pardo River, municipality of Avaré, São Paulo State, Brazil (22° 57′ 48.35″ S; 48° 47′ 26.07″ W).

Site of infection: histozoic, gill filaments (interfilamental type).

Type-material: A glass slide with myxospores (hapantotype) was deposited in the collection of the Instituto Nacional de Pesquisa da Amazônia (INPA), Brazil (Num. INPA93). The ssrDNA partial sequence was deposited in GenBank with accession number OP070162.

Etymology: The specific name is derived from the Latin words: *brevis* (small) and *cauda* (tail) and referred to the small size of their caudal appendages.

Zoobank registration code: urn:lsid:zoobank.org:pub:D6824583-AAD4-4FC8-B67F-40233059FDF2.

Plasmodia: whitish, round and small, measuring about 0.05 mm. Present in the basal region of the gill filaments, occupying an interfilamental position. Average one plasmodia per parasitized fish.

Myxospores: The morphological data are based on the observation of myxospores isolated from a single plasmodium. The myxospores found were elongated and ellipsoidal in frontal view. Two elongated, elliptical shell valves with two small tapering caudal appendages. Average measurements: total length 17.1 ± 0.9 (15.9–18.7) µm, body length 10.7 ± 0.4 (9.6–10.8) µm, body width 5.3 ± 0.5 (4.4–6.1) µm, caudal appendages length 7.2 ± 0.8 (5.8–8.2) µm. Myxospores were biconvex in the lateral view with a thickness of 3.7 ± 0.1 (3.6–3.9) µm. The polar capsules were unequal in size. The larger polar capsule measured 5.4 ± 0.2 (5.1–5.6) µm in length and 1.5 ± 0.1 (1.2–1.6) µm in width, while the smaller polar capsule measured 4.6 ± 0.3 (4.2–5.0) µm in length and 1.2 ± 0.1 (1.1–1.3) µm, occupying about half of the sporoplasm. Seven coils in the polar tubules present inside the polar capsule perpendicular to the longitudinal axis of the polar capsule were observed. Symmetrical valves.

### Molecular and phylogenetic analysis

Three genetically identical partial sequences of the *Henneguya novaerae* n. sp. (1672-bp, 1606-bp, 1604-bp), one partial sequence of *Henneguya bagre* n. sp. (1873-bp), and one partial sequence of *Henneguya breviscauda* n. sp. (1898-bp) from ssrDNA were obtained. The partial sequences were obtained from the type-host of the species. *Henneguya novaerae* n. sp. was 88.2% genetically similar to *Henneguya breviscauda* n. sp. (195 different nucleotides) and 91.4% genetically similar to *Henneguya bagre* n. sp. (162 different nucleotides). *Henneguya breviscauda* n. sp. and *Henneguya bagre* n. sp. are 91.9% genetically similar to each other (134 different nucleotides). The BLAST search showed that the highest similarity to other myxozoan sequences available in GenBank was no higher than 93.6%. Among the species with partial sequences available on GenBank, the one that most genetically resembled the three new species was *H*. *jundiai*. The registered similarity was 93.4% with *Henneguya novaerae* n. sp. (107 different nucleotides), 90.1% with *Henneguya breviscauda* n. sp. (182 different nucleotides), and 93.6% with *Henneguya bagre* n. sp (119 different nucleotides).

**Figure 5:**
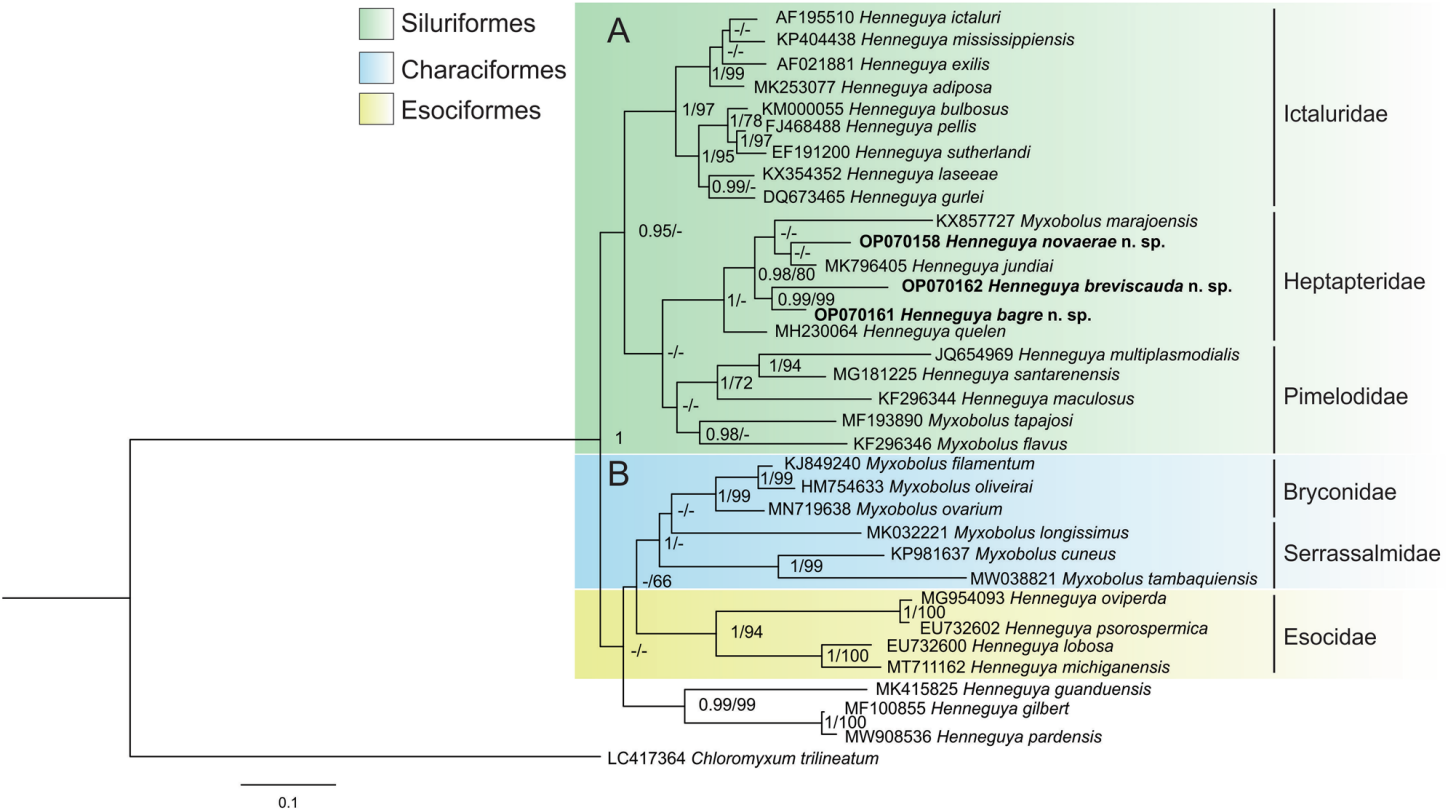
Phylogenetic tree of Bayesian analysis based on partial ssrDNA sequences showing the position of the new species among other genetically similar *Myxobolus*/*Henneguya* species. Node numbers represent the Bayesian posterior probabilities (BI) and bootstrap. Values less than 0.95/65 are represented by dashes. The scale bar represents the number of substitutions per site.

Phylogenetic analysis of species genetically similar to the new species showed a division into two main polyphyletic clades (Fig. 5). Clade A is composed of *Henneguya*/*Myxobolus* spp. that parasitize Siluriformes fishes of the families Ictaluridae, Pimelodidae, and Heptapteridae, where the three new species are inserted. *Henneguya novaerae* n. sp. appears as a sister species of *H*. *jundiai*, while *Henneguya bagre* n. sp. and *Henneguya breviscauda* n. sp. are sister species. All species that integrate the subclade that parasitizes fish of the family Heptapteridae parasitize *R*. *quelen*. Clade B is mainly composed of *Myxobolus* spp. which parasitizes Characiformes fish and *Henneguya* spp. which parasitizes Esociformes fishes.

### Remark

*Henneguya novaerae* n. sp., *Henneguya bagre* n. sp., and *Henneguya breviscauda* n. sp. were distinguished from the other *Henneguya* spp. by the morphological or molecular way. The three new species were compared with each other and with species previously described parasitizing Siluriformes fishes from the Americas (Table 2), and all *Henneguya* species already described, based on the morphometric characteristics of the myxospores.

**Table 2:** Comparative data for the three new species described and *Henneguya* spp. from siluriform fishes from South America.

Species	TL	ML	MW	CA	PL	PW	TH	T	SI	Host
*Henneguya novaerae* n. sp.	24.9 ± 0.9 (23.3–26.8)	10.7 ± 0.5 (10.1–11.6)	3.8 ± 0.3 (3.2–4.2)	14.7 ± 1.4 (11.5–16.1)	4.8 ± 0.4 (3.8–5.5)	1.3 ± 0.2 (1.1–1.7)	3.5 ± 0.3 (3.1–4.0)	6	Gills filaments	*Rhamdia quelen*
*Henneguya bagre* n. sp.	20.9 ± 0.9 (19.2–21.8)	11.2 ± 0.5 (10.5–12.4)	5.1 ± 0.3 (4.6–6.1)	9.8 ± 0.9 (8.2–11.1)	5.9 ± 0.6 (4.9–6.7)	1.6 ± 0.1 (1.4–1.8)	3.8 ± 0.2 (3.6–4.1)	7	Gills filaments	*R*. *quelen*
*Henneguya breviscauda* n. sp.	17.1 ± 0.9 (15.9–18.7)	10.7 ± 0.4 (9.6–10.8)	5.3 ± 0.5 (4.4–6.1)	7.2 ± 0.8 (5.8–8.2)	5.4 ± 0.2 (5.1–5.6)^a^ 4.6 ± 0.3 (4.2–5.0)^b^	1.5 ± 0.1 (1.2–1.6)^a^ 1.2 ± 0.1 (1.1–1.3)^b^	3.7 ± 0.1 (3.6–3.9)	7	Gills filaments	*R*. *quelen*
*H. jundiai*	26.9 ± 1.9 (22.9–29.2)	9.5 ± 0.4 (8.8–10.0)	4.6 ± 0.4 (4.1–5.5)	17.3 ± 1.8 (14.1–19.8)	4.9 ± 0.3 (4.6–5.5)	1.4 ± 0.2 (1.2–1.7)	–	6–7	Gill arches	*R. quelen*
*H. quelen*	40.0 ± 2.8 (37.0–42.8)	15.6 ± 0.8 (14.3–16.4)	4.1 ± 0.3 (3.9–4.4)	24.3 ± 2.2 (21–26.5)	5.5 ± 0.5 (5.2–6.0)	1.6 ± 0.2 (1.4–1.8)	–	–	Kidney	*R. quelen*
*H. rhamdia*	50.0 ± 1.8	13.1 ± 1.1	5.2 ± 0.5	36.9 ± 1.6	4.7 ± 0.4	1.1 ± 0.2	2.5 ± 0.5	10–11	Gill	*R. quelen*
*H. guanduensis*	27.3 ± 38.1	11.4 ± 16.7	4.9 ± 7.9	15.6 ± 22.5	4.4 (3.3–5.6)^a^ 4.1 (3.3–5.3)^b^	2.0 (1.6–2.3)^a^ 2.2 (1.5–2.8)^b^	–	3–6	Gill	*Hoplosternum littorale*
*H. pseudoplatystoma*	33.2 ± 1.9	10.4 ± 0.6	3.4 ± 0.4	22.7 ± 1.7	3.3 ± 0.4	1.0 ± 0.1	–	6–7	Gill filaments	Hybrid *Pseudoplatystoma corruscans*/*P. fasciatum*
*H. eirasi*	37.1 ± 1.8	12.9 ± 0.8	3.4 ± 0.3	24.6 ± 2.2	5.4 ± 0.5	0.7 ± 0.1	3.1 ± 0.1	12–13	Gill filaments	*P. corruscans*; *P. fasciatum*
*H. multiplasmodialis*	30.8 ± 1.3	14.7 ± 0.5	5.2 ± 0.3	15.4 ± 1.3	6.1 ± 0.1	1.4 ± 0.1	4.4 ± 0.1	6–7	Gill	*P. corruscans*
*H. corruscans*	27.6 (25–29)	14.3 (13–15)	5.0	13.7 (12–15)	6.8 (6–7)	2.0	3.1 ± 0.4	5–6	Gill	*P. corruscans*
*H. maculosus*	-	13.7 ± 0.6	4.1 ± 0.2	17.5 ± 1.0	5.6 ± 0.5	1.6 ± 0.2	3.0 ± 0.2	6–7	Gill filaments	*P. corruscans*
*H. cuniculator*	29.4 ± 2.4	12.1 ± 1.0	4.8 ± 0.4	16.7 ± 2.0	6.2 ± 0.3	1.8 ± 0.1	4.2 ± 0.7	10–11	Gill filaments	*P. corruscans*
*H. melini*	40.8 ± 0.3 (40.3–41.1)	15.5 ± 0.2 (15.3–15.7)	4.7 ± 0.1 (4.6–4.8)	25.3 ± 0.1 (25.2–25.4)	4.8 ± 0.5 (4.3–5.3)	1.7 ± 0.3 (1.4–2.0)	–	5–6	Gill filaments	*Corydoras melini*
*H. occulta*	36–46	16–20	8	20	8	–	–	–	Gill	*Loricaria* sp.
*H. pellucida*	33.3 ± 1.5	11.4 ± 0.3	4.1 ± 0.4	24.1 ± 1.5	4.0 ± 0.4	1.6 ± 0.2	–	6–7	Visceral cavity/swin bladder	*Piaractus mesopotamicus*

All measurements are in µm.

TL = total length; ML = myxospore body length; MW = myxospore body width; CA = caudal appendages length; PL = polar capsule length; PW = polar capsule width; TH = thickness; T = number of turns of the polar filament; SI = site of infection.

^a^Larger polar capsules; ^b^Smaller polar capsules.

The three new species described in this study are visually similar and the myxospore body is almost identical in morphology and morphometry. However, there is a clear difference concerning the size of the caudal appendage of the species (14.7 ± 1.4 µm vs. 9.8 ± 0.9 µm vs. 7.2 ± 0.8 µm), which also causes a distinction in the size of the total length of the myxospore (24.9 ± 0.9 µm vs. 20.9 ± 0.9 µm vs. 17.1 ± 0.9 µm). *Henneguya breviscauda* n. sp. also has polar capsules of different sizes, which is not observed in the other two species. *Henneguya novaerae* n. sp. presents the width of the body of the myxospore smaller than *Henneguya bagre* n. sp. (3.8 ± 0.3 µm vs. 5.1 ± 0.3 µm), also differentiating the two species. Furthermore, the molecular differences reported above support the discrimination of the species.

*Henneguya novaerae* sp. was compared with other *Henneguya* spp. that parasitizes *R*. *quelen*. *Henneguya jundiai* is the species that most resembles *Henneguya novaerae* sp. However, there are morphometric differences regarding the length and width of the myxospore body. For *H*. *jundiai* the maximum reported spore body length was 10.0 µm, with an average of 9.5 µm, while for *Henneguya novaerae* n. sp. the measurements found were 10.7 ± 0.5 (10.1–11.6) µm. Concerning the width of the myxospore body, the opposite occurs, with *H*. *jundiai* having larger measurements than those found for *Henneguya novaerae* n. sp. (4.6 ± 0.4 (4.1–5.5) µm vs. 3.8 ± 0.3 (3.2–4.2) µm, respectively). Furthermore, *H*. *jundiai* parasitizes the gill arch of *R*. *quelen*, while *Henneguya novaerae* n. sp. parasitizes the gill filaments. The molecular comparison with 106 different nucleotides (equivalent to 6.6% of the total) in the partial sequences available for the species reinforces the distinction between these two species. About the other two *Henneguya* spp. described parasitizing *R*. *quelen*, *Henneguya novaerae* n. sp. presented smaller measures in almost all characteristics, besides the molecular differences that can be observed in the phylogenetic analysis.

In comparison with species that parasitize Siluriformes fishes from the Americas, the species that most resembled *Henneguya novaerae* n. sp. was *Henneguya pseudoplatystoma* Naldoni, Arana, Maia, Ceccarelli, Tavares, Borges, Pozo & Adriano, 2009. However, *H*. *pseudoplatystoma* presents the length of the caudal appendages greater than those observed in *Henneguya novaerae* n. sp (22.7 ± 1.7 µm vs. 14.7 ± 1.4 (11.5–16.1) µm). *Henneguya novaerae* n. sp. has also been compared to *Henneguya* spp. which parasitizes fishes of other orders and other geographic locations. The species that most resembled *Henneguya novaerae* n. sp. was *Henneguya sharifi* Molnár, Székely, Mohamed & Shaharom-Harrison, 2006, that parasites *Pangasius hypophthalmus* (Sauvage, 1878) from Malaysia. The two species have similar length and width of the myxospore body but differ in the length of the caudal appendages (14.7 ± 1.4 (11.5–16.1) µm vs. 7.5 (5.9–10.3) µm).

The other species described parasitizing Siluriformes from the Americas have a longer caudal appendage concerning *Henneguya bagre* n. sp. and *Henneguya breviscauda* n. sp. *Henneguya corruscans* Eiras, Takemoto and Pavanelli, 2009 is the species that presents the caudal appendages with a size closer (13.7 µm) to that observed in the two new species, but the myxospores body length is greater (14.3 µm), differentiating them.

All other species used for comparison did not show morphological or morphometric characteristics resembling the new species. At least one morphometric feature, the number of turns of the polar tubules (varying at least 3 turns), or partial genetic sequences, differed in comparison with the new species.

## Discussion

In the present study, new species of myxozoans were described based on morphometric characteristics, as well as with phylogenetic support, and the sum of the evidence indicated conclusively the existence of three new species, denominated *Henneguya novaerae* n. sp, *Henneguya bagre* n. sp. and *Henneguya breviscauda* n. sp.

The sequence similarities and variables sites are important evidence for species identification and discrimination of closely related myxozoans species^15,26,27^. Although *H*. *jundiai* has been described in the same main watershed where this study was carried out, geographically close, and morphologically similar, our molecular analyzes showed differences between the referred species and the new species. Furthermore, the preferred location for parasitizing the gills was also different, *H*. *jundiai* parasitizing the gill arches and the new species found in the filaments.

The phylogenetic analysis indicated an affinity of *Henneguya* spp. with the hosts, with a strong evolutionary signal, as reported by Carriero et al.^28^, Vieira et al.^29,30^, and Úngari et al.^31^. In the family Myxobolidae, the hosts are the primary evolutionary factors, which contribute to the few monophyletic clades observed in the phylogenies^32,33^. In this study, the arrangement of the species was determined primarily by the order of the host, followed by the family of the host. The new species are inserted in a polyphyletic clade composed of species that infect Siluriformes fishes, along with other *Henneguya*/*Myxobolus* species that parasitize *R*. *quelen*. The phylogenetic tree clearly distinguishes the new species from the other *Henneguya* spp. deposited in GenBank. This arrangement further corroborates the effectiveness of the ssrDNA gene for analyses of the phylogenetic relationships among myxozoans, given that closely related species can be extremely variable, with their characteristics being determined primarily by the process of coevolution with their respective hosts^34^.

Abrunhosa et al.^25^ and Negrelli et al.^24^ reported the presence of species that parasitize Esociformes fishes in their phylogenetic analyses, as observed in this study. These data corroborate the close phylogenetic relationships between myxozoans species that parasitize fishes from the families Esocidae and Heptapteridae, as only genetically similar species were used for this analysis in this study.

Unfortunately, there is no ssrDNA data available for *H*. *rhamdia* in the GenBank, making it impossible to genetically compare them to the new species or to evaluate the phylogenetic relationships of these species. Future molecular and phylogenetic analysis of *H*. *rhamdia* is highly recommended to enable a definitive comparison and study of the relationship between species.

Differences were observed concerning the prevalence of each myxozoan described in this study about the collection localities of the hosts. *Henneguya novaerae* n. sp. was the only species found at two different localities, being close to the source and mouth of the Pardo River. *Henneguya bagre* n. sp. and *Henneguya breviscauda* n. sp. were only found at a single locality, and the fish parasitized with *Henneguya breviscauda* n. sp. were not parasitized by another myxozoan species. There was co-infection of *Henneguya novaerae* n. sp. and *Henneguya bagre* n. sp. at locality 3. Although the different number of hosts collected per point may have interfered with the prevalence found, it is possible that different populations of *Henneguya* spp. inhabit different populations of *R*. *quelen* along the Pardo River. The various natural and artificial geographic barriers can contribute to this, but future studies are necessary to clarify this statement.

The place of formation of plasmodia is an important taxonomic character for the differentiation of histozoic myxozoan species^35^. Unfortunately, because of the small number of infected hosts, priority was given to molecular identifications rather than histological preparations. Despite this, visual observations and macroscopic photos helped us to determine the position and shape of plasmodia in the gills. Future studies should determine which tissue of the gill is the specific site of infection for the new species.

The present study describes *Henneguya novaerae* n. sp., *Henneguya bagre* n. sp., and *Henneguya breviscauda* n. sp., gill parasites of *R*. *quelen* from Brazil. This is an important contribution to the understanding of the microparasite fauna of the siluriform species of the Neotropical region. The study indicates too that molecular characteristics and microhabitat preferences in the hosts are important for distinguishing morphologically similar species. Based on our analysis we recommend monitoring the presence of these parasites in farmed fish, to analyze possible pathologies caused by them.

## Material and methods

### Host sampling and parasitological procedures

Specimens of *R*. *quelen* were collected from three localities in Pardo River: a region near the river source (locality 1), municipality of Botucatu, Nova Era Resort (coordinates 22° 58′ 40.03″ S; 48° 30′ 51.53″ W) in June 2021; intermediate region (locality 2), municipality of Avaré (coordinates 22° 57′ 48.35″ S; 48° 47′ 26.07″ W) in January 2022; and a region near the river mouth (locality 3), municipality of Salto Grande (coordinates 22° 54′ 15.90″ S; 49° 57′ 06.5″ W) in May 2022. The collection was authorized by the Instituto Chico Mendes de Conservação da Biodiversidade (SISBIO #60640–1). Fish were collected using casting nets and were euthanazied with sodium thiopental (Thiopentax®), measured, and examined. All procedures followed the recommendations of the Ethical Commission for Animal Experimentation from the São Paulo State University (Unesp), Institute of Biosciences, Botucatu, Brazil (CEUA n^o^ 9415260520). The experimental protocol was approved by Ethical Commission for Animal Experimentation from the São Paulo State University (Unesp), (CEUA n^o^ 9415260520). The study is reported in accordance with ARRIVE guidelines. Fish were captured using a seine net with a small mesh (mosquito net fishing), cast nets, and a hand net. The voucher specimen of the fish host was fixed in 10% formalin, and deposited in Brazilian fish collection: Laboratory of Fish Biology and Genetics of the São Paulo State University (UNESP), São Paulo State (LBP 32004). Host specimens were examined fresh and the internal and external organs were evaluated in search of plasmodia containing myxospores using a Leica S6 D stereomicroscope (Leica Microsystems, Germany) with a 16 × ocular. Samples of gills infected with plasmodia were collected for morphological and molecular analysis. The prevalence of parasites was calculated according to Bush et al.^36^. The morphological measurements of the fresh myxospores followed the recommendations of Lom and Arthur^37^. The measurements of 30 myxospores were performed using a computerized image analysis system with differential interference contrast (DIC) (Leica Application Suite, V3; Leica Microsystems, Wetzlar, Germany). Morphometric data were obtained from myxospores from one plasmodium of the type-host. All measurements are presented in micrometers (μm) and are expressed as means, followed by the standard deviation (SD), and range in parentheses. Illustrations of the myxospores were produced with the aid of a camera lucida mounted on a Leica DMLS microscope with phase-contrast optics. The final images were edited using CorelDRAW X8 software (Corel Corporation, Canada).

### DNA extraction, amplification, sequencing, and alignments

Samples containing plasmodia were removed from the gills and fixed in absolute ethanol (100%). Genomic DNA was extracted from the species using the DNeasy® Blood & Tissue Kit (Qiagen, Valencia, CA, USA), following the manufacturer’s protocol. At least one of the samples used to obtain the partial sequences came from the type-host species. Access to the genetic data was authorized by the Brazilian Ministry of Environment (Sisgen A8A1D2B). Fragments of ssrDNA were amplified using the primers and cycling conditions presented in Table 3. Conventional polymerase chain reaction (PCR) amplifications were performed on a final volume of 25 µL using ready-to-go PCR beads (GE Healthcare) with extracted DNA (3.0 µL) and 1.0 µL of each PCR primer. PCR products (2.0 µL) were run on an agarose gel (1%) using GelRed™ fluorescent nucleic acid dye and loading buffer to confirm amplicon size and yield. The PCR reaction products for the ssrDNA gene were purified with magnetic beads from the Ampure XP kit (Beckman Coulter) following the manufacturer’s protocol and sequenced using the same set of PCR amplification primers. Automated sequencing was performed directly on purified PCR products using a BigDye v.3.1 Terminator Cycle Sequencing Ready Reaction kit on an ABI 3500 DNA genetic sequencer (Applied Biosystems). The new sequences were assembled and edited using Sequencher v. 5.2.4 (Gene Codes, Ann Arbor, MI, USA). The similarity of the partial sequences obtained with other sequences available in the GenBank database was obtained through searches in the basic local alignment search tool (BLAST). The species most genetically similar (> 80%) to the new species were included in the phylogenetic analyses. The alignments were performed using the MUSCLE algorithm implemented on Geneious 7.1.3^38^ with default settings.

**Table 3:** Primers used for the amplification and sequencing of the ssrDNA of the new *Henneguya* spp.

Primer	Sequence 5′–3′	Paired with	Reference
Erib1	ACCTGGTTGATCCTGCCAG	Act1r	Barta et al.^39^
Myxgen4F	GTGCCTTGAATAAATCAGAG	Erib10	Kent et al.^40^
Act1r	AATTTCACCTCTCGCTGCCA	Erib1	Hallett and Diamant^41^
Erib10	CTTCCGCAGGTTCACCTACGG	Myxgen4F	Barta et al.^39^
MB5	GGTGATGATTAACAGGAGCGGT	MX3	Eszterbauer^42^
MX3	CCAGGACATCTTAGGGCATCACAGA	MB5	Andree et al.^32^

Cycling conditions (for all primers): Initial denaturation at 95 °C for 3 min, followed by 35 cycles of 95 °C for 1 min, annealing at 55 °C for 45 s, 72 °C for 2 min, and a final extension at 72 °C for 7 min.

### Phylogenetic analyses

The best-fitting models for nucleotide substitution selected by jModelTest software^43^ using the Akaike information criterion was GTR + I + G. Phylogenies were reconstructed for each alignment under Bayesian inference (BI) using MrBayes v. 3.2^44^. and Maximum Likelihood (ML) using PhyML^45^ with 1000 replicates. The BI was run using four Markov chain Monte Carlo searches with 10,000,000 generations and sampling tree topologies every 100 generations. The burning was set to the first 30% of generations; the consensus trees were estimated using the remaining topologies. The nodes with posterior probabilities and bootstrap values greater than 0.95/65 were considered valid. The final trees were edited using CorelDRAW X8. Pairwise genetic distances within and among sequences were calculated using the Kimura-2-parameter (K2P) model and a bootstrap procedure with 1000 replicates in Geneious 7.1.3^38^.

## Data Availability

The datasets generated and/or analyzed during the current study are available in the GenBank repository, codes OP070158-OP070162. Permanent access links: https://www.ncbi.nlm.nih.gov/nuccore/OP070158, https://www.ncbi.nlm.nih.gov/nuccore/OP070159, https://www.ncbi.nlm.nih.gov/nuccore/OP070160, https://www.ncbi.nlm.nih.gov/nuccore/OP070161, https://www.ncbi.nlm.nih.gov/nuccore/OP070162.
